# Rare Gastroesophageal Tumor Subtypes: Clinicopathologic Characteristics, Molecular Alterations, and Therapeutic Implications

**DOI:** 10.3390/cancers18142210

**Published:** 2026-07-09

**Authors:** Fatemeh Sadat Tabatabaei, Nicholas J. Caldwell, Nattaya Teeyapun, Seyed Mohammad Amin Dashti, Sienna M. Durbin, Matthew Strickland, Jonathan N. Glickman, Samuel J. Klempner

**Affiliations:** 1 Center for Immunology and Inflammatory Diseases, Massachusetts General Hospital, Mass General Brigham, Boston, MA 02129, USA; ftabatabaei@mgh.harvard.edu; 2 Harvard Medical School, Boston, MA 02115, USA; 3 Department of Pathology, Mass General Brigham, Boston, MA 02114, USA; njcaldwell@mgh.harvard.edu; 4 Division of Medical Oncology, Department of Medicine, Faculty of Medicine, Chulalongkorn University, Bangkok 10330, Thailand; amprabbit@yahoo.co.th; 5 School of Medicine, Tehran University of Medical Sciences, Tehran 1416753955, Iran; amindashti2329@gmail.com; 6 Department of Surgery, Tehran University of Medical Sciences, Tehran 1416753955, Iran; 7 Division of Hematology-Oncology, Mass General Brigham Cancer Institute, Boston, MA 02114, USA; sienna_durbin@dfci.harvard.edu (S.M.D.); mstrickland1@mgh.harvard.edu (M.S.)

**Keywords:** gastroesophageal cancers, biomarkers, immune checkpoint inhibitors, precision oncology, rare subtype

## Abstract

Rare subtypes of gastroesophageal malignancies represent a small but biologically meaningful fraction of upper gastrointestinal cancers. Although most therapeutic algorithms are derived from conventional squamous cell carcinoma and adenocarcinoma, uncommon entities such as variants of squamous cell carcinoma, lymphoepithelioma-like carcinoma, adenosquamous carcinoma, neuroendocrine carcinoma, and others display distinct clinicopathologic, immunologic, and molecular features that may influence prognosis and therapeutic decision-making. This review synthesizes current evidence regarding the epidemiology, histopathology, molecular alterations, and emerging therapeutic vulnerabilities across these rare subtypes. Importantly, these tumors frequently exhibit aggressive clinical behavior and are often managed by extrapolation from more common histologies due to the absence of prospective data. Increasing integration of genomic profiling, immune characterization, and biomarker-driven stratification is essential to refine diagnoses, expand precision therapeutic strategies, and improve outcomes. Recognition of these rare subtypes in routine practice is critical, as even small molecularly defined populations may carry disproportionate biological and translational significance within oncology.

## Simple Summary

The vast majority of gastroesophageal cancers are classified as either squamous cell carcinoma or adenocarcinoma, and current treatment strategies are informed by these broad classifications. However, several rare tumor subtypes arise in the esophagus and stomach that differ in their histomorphologic appearance, behavior, biomarker profiles, and response to therapy. Because these subtypes are uncommon, they are often underrecognized and managed using treatment approaches designed for more common cancers. In this review, we summarize the key clinical, pathologic, and molecular features of rare gastroesophageal tumor subtypes. Greater awareness of these rare entities may improve diagnosis, support more personalized treatment decisions, and ultimately enhance patient outcomes.

## 1. Introduction

Gastric, esophageal, and gastroesophageal junction (GEJ) malignancies constitute a major global health burden. In 2020 alone, these cancers were responsible for more than one million newly diagnosed cases and approximately 769,000 deaths, ranking as one of the most common cancers worldwide and among the leading causes of cancer-related mortality [1]. The vast majority of gastric malignancies are adenocarcinomas, whereas primary gastric squamous and other non-adenocarcinomatous carcinomas are rare [2]. The majority of esophageal cancers are squamous cell carcinomas (SCC); however, the incidence of esophageal adenocarcinoma has shown a steady rise in recent decades [3]. Diagnosis is established through upper endoscopy with biopsy, followed by histopathologic evaluation and appropriate staging using various imaging modalities. Histopathologic assessment remains the cornerstone of diagnosis, while molecular characterization has become increasingly important for therapeutic decision-making. Clinically relevant biomarkers include HER2 overexpression or amplification (primarily in adenocarcinomas), PD-L1 expression, and microsatellite instability (MSI) or mismatch repair deficiency (dMMR). Treatment strategies include endoscopic resection for selected early-stage lesions, surgery with perioperative or adjuvant therapy for resectable disease, chemoradiotherapy for appropriate patients, and systemic chemotherapy, targeted therapy, and immune checkpoint inhibitors for advanced or metastatic disease [1,2].

Beyond these two predominant histologic subtypes, the gastroesophageal mucosa can give rise to a diverse array of uncommon neoplasms, each exhibiting distinct histopathologic characteristics and varying prognostic implications and molecular landscapes. In this review, we bring together the collection of rare gastroesophageal cancer subgroups and place them in the context of modern therapeutic approaches.

## 2. Variants of Squamous Cell Carcinoma

Esophageal squamous cell carcinoma accounts for approximately 80% of all esophageal cancers globally [4]. Its incidence shows marked geographic variation, with particularly high rates reported in East Asia, eastern and southern Africa, and parts of southern Europe [5]. Established risk factors include tobacco use and alcohol consumption, which act synergistically to increase disease risk, along with genetic and environmental influences [6].

### 2.1. Esophageal Verrucous Squamous Cell Carcinoma

Esophageal verrucous squamous cell carcinoma (VSCC) is an uncommon well-differentiated variant of SCC characterized by deceptively bland histology but potentially devastating local behavior [7]. Since the first esophageal description in 1967, fewer than 100 and, in several summaries, fewer than 50 cases have been reported worldwide [8]. Patients are typically between 50 and 80 years, a mean age around 67 years, and male predominance of approximately 2:1 to 3:1 [7,8]. Cases have been described across Asia, Europe, and North America without a clear geographic cluster [8]. Lesions most commonly involve the middle and distal esophagus, and tumors may show extensive superficial spread, with reported lengths ranging from 2 to 22 cm and a median of 9 cm [9].

Clinically, VSCC differs from routine esophageal squamous cell carcinoma (ESCC) less by symptom type than by tempo [7]. Dysphagia is the dominant presentation and has been reported in more than 80% of cases, but progression is often insidious over months to years rather than rapidly progressive [7,8]. Weight loss and odynophagia are frequent, and a particularly important clue is refractory candidiasis or persistent white plaque-like mucosal disease that does not resolve with antifungal therapy [7,9]. In advanced cases, cough, chest pain, or airway symptoms may reflect local extension or fistula formation [9]. This slow but relentless course explains why many patients are diagnosed late despite prolonged evaluation [8,10].

Diagnosis remains the major clinical challenge. On endoscopy, VSCC may appear as a white plaque-like lesion, a verrucous or wart-like exophytic mass, or diffuse mural thickening [7,9]. Superficial biopsies are frequently nondiagnostic because the tumor surface is dominated by hyperkeratosis, acanthosis, inflammation, and superimposed Candida, whereas cytologic atypia may be minimal [9]. Several reports emphasize that repeated superficial sampling can delay definitive diagnosis for many months; in one report, confirmation was obtained only 9–10 months after initial presentation [8]. For this reason, persistent suspicious lesions require a low threshold for deep biopsy, diagnostic Endoscopic Mucosal Resection/Endoscopic Submucosal Dissection (EMR/ESD), or resection-based sampling [9]. Endoscopic ultrasound (EUS) can help define local invasion and nodal status, and narrow-band imaging may assist in margin delineation in early lesions [7,11].

**Figure 1:**
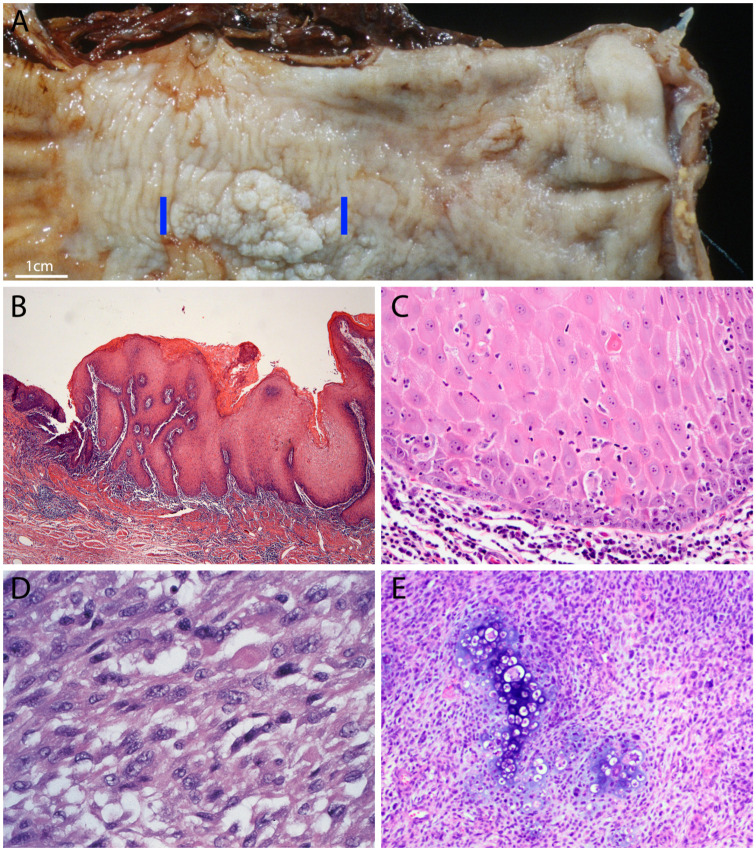
Representative photographs of verrucous carcinoma (**A**–**C**), spindle cell squamous cell carcinoma (**D**) and carcinosarcoma (**E**). (**A**) Gross photograph of esophagectomy (proximal esophageal margin right, gastroesophageal junction left) for verrucous carcinoma (flanked between two blue bars), showing exophytic plaque-like tumor in the distal esophagus (1 cm scale bar at bottom left of image). (**B**) Low magnification view of verrucous carcinoma, showing a well-differentiated squamous proliferation with a pushing lower border (H&E, original image at 20× mag.). (**C**) High magnification view of deep aspect of verrucous carcinoma. Note well-differentiated squamous cells with minimal nuclear atypia and low nuclear cytoplasmic ratio (H&E, original image at 200× mag.). (**D**) High magnification view of spindle cell squamous cell carcinoma showing cytologic features of spindle cells, with nuclear pleomorphism and vesicular chromatin (H&E, original image at 400× mag.). (**E**) High magnification view of esophageal carcinosarcoma showing area of tumor with heterologous chondroid differentiation (H&E, original image at 200× mag.). Legend: mag., magnification.

Histologically, VSCC shows marked keratinization, a church-spire or verruciform surface contour, and broad pushing borders rather than the infiltrative nests typical of conventional ESCC [8,11] (Figure 1A–C). The molecular profile also appears distinct from routine ESCC [12]. In the available summaries, p16 immunohistochemistry is usually negative, p53 immunohistochemistry often shows a wild-type or non-mutant pattern, and HPV does not appear to play a consistent etiologic role [13]. Recent work suggests *SMARCA4* alterations may be frequent in VSCC, with some series reporting abnormalities in more than 80% of cases, raising the possibility that molecular testing may assist difficult diagnostic distinctions [12]. Interestingly, *TP53* mutation has been identified only in one metastatic case. The *TP53*-mutated VSCC harbored a heterozygous point mutation in position c.738G > A of exon 7 of *TP53*, resulting in the mutant variant p.M246I [13].

Prognosis is determined less by distant dissemination than by the consequences of delayed diagnosis and uncontrolled local invasion [9,14]. Lymph node metastasis is often absent, but broader series suggest nodal involvement may occur in up to 11.1% of cases. Distant metastasis is rare [7,9]. Historically, mortality rates as high as 67% were reported, largely because patients died from local complications such as stenosis, fistula, aspiration, or airway invasion rather than from widespread metastatic disease [14]. By contrast, when diagnosis is established early and treatment is definitive, recurrence appears uncommon and disease-free survival can be prolonged [9,11,15].

Management is stage-dependent but remains driven primarily by local control. For localized or resectable disease, esophagectomy with lymph node dissection remains the standard approach and offers the most reliable chance of cure. Modern surgical reports describe excellent long-term outcomes, including disease-free follow-up beyond 7 to 8 years [9]. In early mucosal lesions, endoscopic resection may preserve the esophagus. Both ESD and EMR have been successfully used in small superficial tumors, with no recurrence reported in selected patients during follow-up [11,15]. Case report data suggests that historical assumptions about universal radioresistance are too simplistic and complete responses are reported with concurrent cisplatin/5-FU with radiation [16]. No established role currently exists for targeted therapy or immune checkpoint inhibition, and the available literature does not provide convincing efficacy data for either strategy in VSCC [7,12].

Taken together, VSCC should not be mistaken for an indolent lesion simply because it is histologically well-differentiated [9,14]. Its key clinical lesson is that deceptively bland morphology may coexist with highly morbid local behavior, making aggressive diagnostic sampling and timely local therapy essential [8,9] (Table 1).

### 2.2. Spindle Cell Squamous Cell Carcinoma

Spindle cell squamous cell carcinoma (SCSCC) is an uncommon histologic variant of conventional SCC, characterized by a variably cellular sarcomatoid spindle cell component. Histopathologic examination typically reveals a biphasic architecture composed of varying proportions of conventional squamous epithelial elements intermingled with pluripotent spindle cells [17] (Figure 1D). Dedicated epidemiologic studies focusing exclusively on esophageal SCSCC are lacking. It accounts for approximately 0.5% to 2.4% of all esophageal neoplasms. Available case series suggest a demographic distribution similar to conventional ESCC, with a predominance in older males. No distinct risk factors unique to SCSCC have been identified, and established SCC risk factors, including tobacco and alcohol exposure, are presumed to play a contributory role [101].

Immunohistochemical profiling reveals cytokeratin positivity in the squamous epithelial component, whereas the spindle cell areas demonstrate heterogeneous staining for cytokeratin, vimentin, and smooth muscle actin. The parallel distribution of p53 protein across both cellular elements indicates a likely clonal relationship. Additionally, altered expression of adhesion molecules, most notably the reduction or absence of E-cadherin, appears to be linked with the morphological transition of squamous tumor cells into a spindle phenotype, likely reflective of cellular plasticity [18]. Transcriptomic and functional studies have shown that Epithelial–mesenchymal transition (EMT) involves coordinated repression of epithelial differentiation programs, cytoskeletal remodeling, and acquisition of mesenchymal features, and is increasingly viewed as a dynamic spectrum rather than a binary state [102]. Roma et al. performed a comprehensive component-resolved multi-omics analysis of pulmonary pleomorphic carcinoma. Transcriptomic analyses revealed that the sarcomatoid/spindle component was characterized by suppression of epithelial differentiation and cell-adhesion programs alongside enrichment of EMT-associated pathways, cytoskeletal reorganization, and cell motility signatures [103]. Methylation analyses further supported epigenetic reprogramming accompanying these transcriptional changes. Yang and colleagues [104] conducted an integrated molecular analysis of 56 pulmonary sarcomatoid carcinomas. Genomic analyses revealed extensive shared trunk mutations between components, supporting a model of clonal evolution from a common epithelial progenitor. Evolutionary reconstruction indicated that sarcomatoid differentiation arises as a later event during tumor progression rather than from an independent lineage. By contrast, spindle cell–specific transcriptomic data, including RNA sequencing, remain lacking for gastroesophageal SCSCC. Extrapolating from other tumor types, it is plausible that similar EMT-associated transcriptional programs may contribute to spindle cell differentiation in this setting; however, this hypothesis requires direct validation.

The standard management involves a multimodal treatment strategy, shared with management of traditional ESCC. Post-treatment outcomes indicate that the 5-year survival rate for SCSCC does not differ significantly from that of conventional SCC [105]. In summary, SCSCC represents a rare sarcomatoid variant of SCC characterized by biphasic morphology and EMT-associated phenotypic plasticity. While its clinical behavior and management largely parallel conventional SCC, the absence of tumor-specific molecular data highlights an important gap that warrants dedicated genomic and transcriptomic investigation (Table 1).

### 2.3. Carcinosarcoma

Within this sarcomatoid spectrum, carcinosarcoma represents a closely related biphasic subtype characterized by more prominent mesenchymal differentiation. Histologically, carcinosarcoma is defined by the presence of both epithelial (carcinomatous) and mesenchymal (sarcomatous) elements (Figure 1E). Carcinosarcoma is currently regarded by recent classifications as part of the spindle cell/sarcomatoid SCC spectrum rather than a completely separate entity [19].

Esophageal carcinosarcoma (ECS) is rare, comprising 0.5–2.4% of malignant esophageal tumors [20]. It is most frequently observed in older male patients, often associated with tobacco and alcohol use and sometimes occurring in the context of preexisting SCC of the esophagus [19]. A recent institutional series analyzing over 40 cases reported that ECS arises predominantly in the middle (≈44%) and lower (≈50%) esophagus, whereas upper-esophageal involvement is rare. Gross morphology varies across polypoid, ulcerative, and medullary patterns, suggesting diverse modes of growth and possible diagnostic challenges during endoscopy [21].

Morphological studies have identified transition zones between carcinomatous and sarcomatous regions, with both components sharing identical genetic alterations, supporting a common clonal origin [22]. Emerging molecular analyses demonstrate that the EMT pathway may play a key role in sarcomatous transformation. In particular, the EMT-associated transcription factor ZEB1 has been identified as a differentially expressed molecular target, showing strong upregulation in the sarcomatous component and implicating ZEB1-mediated EMT in tumor progression and phenotypic conversion [23].

There are no large-scale genomic or transcriptomic studies, such as whole-exome sequencing or RNA sequencing, systematically reported for this entity. Anecdotal data from whole-genome sequencing in two patients revealed recurrent driver events shared between both cases, including *TP53* mutations and copy number gains at 11q13 encompassing *CCND1*. Additional copy number gains affecting oncogenic pathways were identified, such as amplifications involving *PIK3CA* and *RICTOR*, implicating activation of the PI3K/Akt/mTOR and cell-cycle signaling pathways. Structural genomic alterations, including chromosomal doubling and chromothripsis-like patterns, were observed, suggesting marked genomic instability. Notably, novel fusion events were detected, including a *TTC28*::*MECOM* fusion in one case, validated by PCR. Germline analysis further identified truncating pathogenic variants in Fanconi anemia pathway genes (*FANCI* and *FANCG*), implicating defective DNA damage repair mechanisms in tumor development [106].

The overall prognostic outlook for ECS remains uncertain. Because the tumor usually displays a polypoid configuration and tends not to invade deeply into the esophageal wall, patients often experience dysphagia early, leading to earlier diagnosis and, in some reports, better survival than those with SCC of comparable size [107,108,109]. However, Sano et al. observed that among T1-stage tumors, the 5-year survival was significantly lower in ECS than ESCC (47.6% vs. 84.3%; *p* = 0.008), likely due to hematogenous spread of the sarcomatous element rather than lymphatic dissemination [24]. Conversely, Wang et al. evaluated 33 ECS cases and reported a superior 5-year survival rate compared to ESCC (48% vs. 34.2%) and a 2-year progression-free survival (PFS) of 50%, which plateaued thereafter [25]. Similarly, Chen et al. analyzed 24 ECS patients and found a 5-year survival rate of 54.2%, seemingly higher than ESCC [26]. In that cohort, 42% of patients had stage T1 disease without nodal involvement, achieving an exceptionally favorable 90% 5-year survival, whereas lymph-node metastases were observed in 57% of T2–T4 tumors, indicating that multimodal therapy may be necessary for advanced stages [26]. Interestingly, patients treated with chemoradiotherapy or radiotherapy alone demonstrated a 5-year survival of 60%, implying that some tumors respond well to non-surgical modalities [26]. Because of its low incidence, there is no universally accepted treatment standard, and current management typically follows that of conventional esophageal carcinoma [110]. Only a few small case series have explored treatment approaches and patient outcomes for ECS [24,25,26], all of which emphasize esophagectomy as the main curative option. While occasional cases have achieved a pCR to chemoradiation [111,112], these remain exceptions. Given the relatively high incidence of lymph node metastasis in advanced-stage disease, multimodal treatment incorporating systemic chemotherapy with or without radiotherapy should be considered in appropriately selected patients.

In conclusion, ECS is a rare biphasic malignancy within the spindle cell/sarcomatoid SCC spectrum with heterogeneous clinical behavior and an uncertain prognosis, likely influenced by EMT-driven tumor evolution. Given the lack of comprehensive genomic and transcriptomic profiling, future molecular studies are essential to further clarify its biology (Table 1).

## 3. Lymphoepithelioma-like Carcinoma

Lymphoepithelioma-like carcinoma (LELC); also known as medullary carcinoma or, if in the stomach, gastric carcinoma with lymphoid stroma (GCLS); is characterized by a prominent intratumoral lymphocytic infiltrate and variable rates of EBV infection. GCLS accounts for approximately 1% to 4% of all gastric cancers. More than 80% of GCLS cases are associated with Epstein–Barr virus (EBV) infection [113]. According to Carrasco-Avino et al. the prevalence of GCLS is higher in American countries than in Asian regions [114], and the incidence among men is roughly twice that of women [115]. Because of the low diagnostic yield of forceps biopsy [27], most cases are diagnosed only after larger sampling methods including ESD. It very rarely occurs in the esophagus; up to 2023, only 39 cases have been reported [28]. The median age at diagnosis was 68.5 years (range, 45–79 years), with a male predominance (75%). The middle thoracic esophagus was the most common site (65%), followed by the lower thoracic (12.5%) and upper thoracic (10%) regions. Most patients (62.5%) presented with advanced disease (Stage II or higher) at diagnosis [116]. Notably, EBV positivity has been detected in 6 cases among all reported esophageal LELCs though a causal relationship is not known [29].

**Figure 2:**
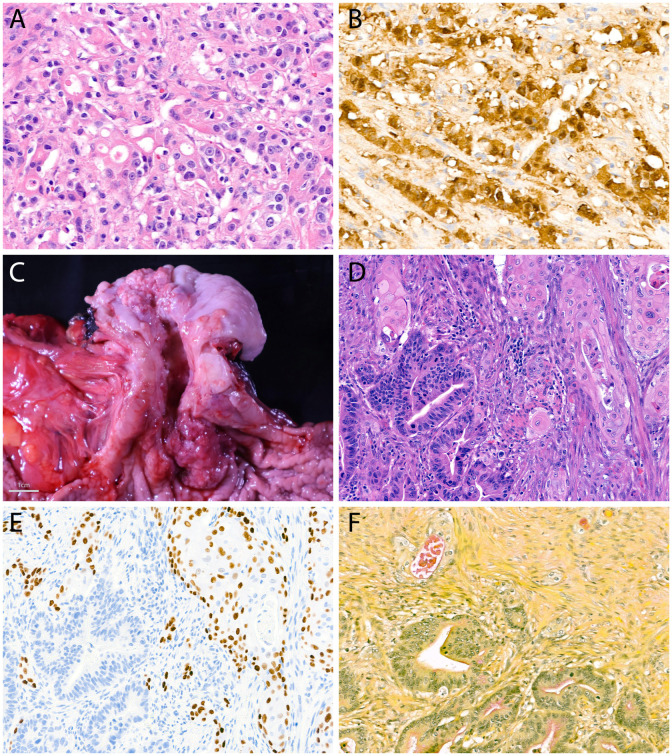
Representative photographs of lymphoepithelioma-like carcinoma (**A**,**B**) and adenosquamous carcinoma (**C**–**F**). (**A**) This gastric lymphoepithelioma-like carcinoma is composed of irregular trabeculae and syncytia of polygonal cells within a conspicuous background of lymphoid cells. (H&E, original photo at 200× mag.). (**B**) The tumor shows diffuse signal with EBER in situ hybridization (EBER ISH, original photo at 200× mag.). (**C**) An opened esophagogastrectomy specimen (proximal esophageal margin top center) demonstrating a circumferential tumor (adenosquamous carcinoma) at the gastroesophageal junction (1 cm scale bar at bottom left of image). (**D**). Microscopically, the tumor demonstrates both an adenocarcinoma component (left) and squamous cell carcinoma component (right) (H&E, original photo at 200× mag.). (**E**) The squamous cell carcinoma component is positive for p40 (p40 IHC, original photo at 200× mag.). (**F**) In a different focus, focal mucin production is present (mucicarmine stain, original photo at 200× mag.). Legend: IHC, immunohistochemistry; ISH, in situ hybridization; mag., magnification.

From a histopathologic standpoint, LELC is composed of irregular trabeculae and syncytia of polygonal cells with a characteristic findings being the presence of a dense intratumor lymphocytic infiltrate (Figure 2A,B). The tumor does have some morphologic overlap with a poorly differentiated non-keratinizing SCC, particularly with reports of immunohistochemical positive for high molecular weight keratins and p40/p63 [117].

In GCLS, Gullo et al. analyzed the tumor microenvironment and found that tumors often contain a cytotoxic T-cell–rich infiltrate at the invasive front, suggesting a potential immune-mediated protective role [30]. Recent studies have also shown that GCLS exhibits higher PD-L1 expression than conventional gastric carcinomas [31,32]. The frequent coexistence of EBV infection and PD-L1 overexpression in LELC suggests that these tumors may respond favorably to immunotherapy, although direct evidence remains limited [118].

Available data regarding treatment of localized esophageal LELC is limited to case reports and small series, although surgical resection following neoadjuvant therapy has been reported to have favorable outcomes. Esophageal LELC appears to be sensitive to chemoradiotherapy and platinum-based chemotherapy, with several reports describing complete responses or marked tumor regression [28,115,116,119]. No prospective data are currently available to guide management.

In advanced disease, data on the treatment of LELC of both the stomach and esophagus remain limited. In most cases, there are no disease-specific treatment guidelines, and management is generally extrapolated from standard regimens used for conventional gastric or esophageal adenocarcinoma, without a clearly defined unique therapeutic approach for LELC. In retrospective reports, the majority of patients received platinum-based chemotherapy as first-line treatment with overall response rate and disease control reported at 46.9% and 83.2%, respectively [120].With regard to immunotherapy, there is a biological rationale for the use of immune checkpoint inhibitors, particularly in tumors with high PD-L1 expression. In addition, EBV-associated LELC, which is also observed in GCLS, may represent a more immunogenic subtype and therefore potentially more responsive to immunotherapy [31,121].

In summary, lymphoepithelioma-like carcinoma of the gastrointestinal tract represents a rare entity with distinct clinicopathologic and immunologic features and a generally favorable prognosis compared with conventional carcinomas. However, the absence of dedicated genomic and transcriptomic data underscores the need for future molecular studies (Table 1).

## 4. AFP-Producing Carcinomas

Alpha-fetoprotein (AFP)–producing gastrointestinal carcinomas have been predominantly described in the stomach, with only a limited number of esophageal or GEJ carcinomas reported to date [44]. The overall frequency of AFP-producing esophageal and GEJ carcinomas (0.7%) parallels that of AFP-producing gastric carcinomas (0.3–2%) recognized in the 2019 World Health Organization (WHO) classification [122].

Motoyama et al. categorized AFP-producing carcinomas into three morphologic categories, the hepatoid type, yolk sac tumor–like type, and fetal gastrointestinal type [123], whereas Kinjo et al. proposed a broader classification including the common adenocarcinoma type, enteroblastic type, hepatoid type, and yolk sac tumor type [45]. The fetal gastrointestinal type and enteroblastic type are now regarded as equivalent designations for adenocarcinoma with enteroblastic differentiation [46]. In gastric lesions, the surface mucosa typically contains tubular adenocarcinoma, whereas the deeper mucosal and submucosal layers show a mixture of tubular adenocarcinoma and areas with enteroblastic differentiation [124]. According to Kinjo et al., AFP-producing carcinomas likely arise from pre-existing tubular adenocarcinoma and subsequently invade into deeper tissue layers [45].

### 4.1. Hepatoid Adenocarcinoma

Hepatoid adenocarcinoma (HAC) is a rare and highly aggressive extrahepatic adenocarcinoma that morphologically and functionally resembles hepatocellular carcinoma (HCC) [33]. Population-based analysis estimated an overall incidence of 0.014 per 100,000 between 2000 and 2016, rising to 0.025 per 100,000 by 2016 [34]. The median age at diagnosis is 66 years, with a reported range of 31–85 years, and SEER data showed a near-equal overall sex distribution; however, gastric hepatoid adenocarcinoma (HAS) demonstrates clear male predominance, with 75.1% of cases occurring in men [34]. Clinical series arise predominantly from East Asia, especially China and Japan, where registry-based datasets identify the lung as the most common registered primary site, while the clinical literature still supports the stomach as the dominant site of origin [33,34,35].

**Figure 3:**
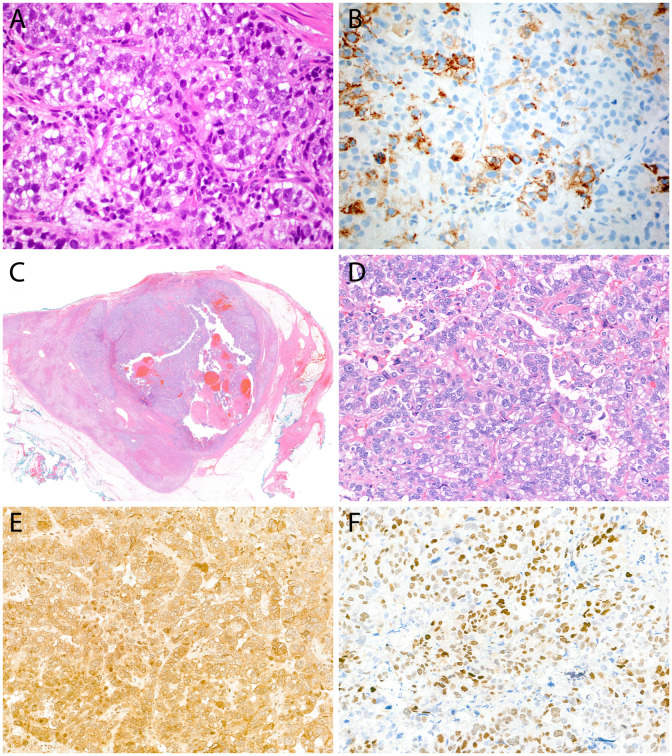
Representative photographs of hepatoid adenocarcinoma (**A**,**B**) and adenocarcinoma with enteroblastic differentiation (**C**–**F**). (**A**) This hepatoid adenocarcinoma is composed of polygonal cells with variable cytoplasmic clearing arranged in sheet-like and trabecular architecture. (H&E, original photo at 200× mag.). (**B**) The tumor shows patchy expression of HepPar-1 (HepPar-1 IHC, original photo at 200× mag.). (**C**) This low power microphotograph demonstrates metastatic adenocarcinoma with enteroblastic differentiation involving an adrenal gland (H&E, original photo at 6× mag.). (**D**) Higher magnification shows tumor cells with cytoplasmic vacuolization (H&E, original photo at 200× mag.). (**E**) The tumor stains for glypican-3 (glypican-3 IHC, original photo at 200× mag.). (**F**)The tumor is also positive for SALL4 (SALL4 IHC, original photo at 200× mag.). Legend: IHC, immunohistochemistry; mag., magnification.

HAC is histologically characterized by hepatoid morphology with large polygonal cells, abundant eosinophilic cytoplasm, and trabecular or sheet-like growth patterns [125] (Figure 3A,B). Rare regions of more conventional adenocarcinoma morphology may be seen. Hepatoid differentiation can be confirmed with immunostains for HepPar-1, glypican-3, and arginase [36]. Given HAC’s large morphologic and immunophenotypic overlap with HCC, a major diagnostic challenge is differentiating a primary esophagogastric HAC from metastatic HCC of the liver, particularly when liver lesions are present [33,37,126]. Detection of a portion of tumor with morphology resembling conventional adenocarcinoma could support a diagnosis of HAC. SALL4, BSEP, CDX2, and MDR3 immunohistochemistry may be useful in this distinction, with studies showing high specificity for these markers for HAC [127,128,129]. Serum AFP is elevated in 76.7–87.1% of patients, and pre-treatment AFP levels above 300–500 ng/mL correlate with inferior survival [38,39]. Recent genomic studies have further characterized the molecular landscape of hepatoid adenocarcinoma. Whole-exome sequencing identified *TP53* as the most frequently mutated gene (66%), with recurrent alterations involving DNA repair and p53 signaling pathways. In addition, mutational signature analysis suggested defective homologous recombination-related DNA damage repair as a potential mechanism contributing to tumorigenesis. Clinically actionable genomic alterations, including amplifications of ERBB2, FGFR1, EGFR, MET, CDK4, and MDM2, as well as *BRCA1/2* mutations, were identified in more than half of the cases, highlighting potential opportunities for biomarker-guided targeted therapies [130].

The biological behavior of HAC is dominated by vascular invasion, nodal spread, and a marked tendency for liver metastasis [33,39]. In a population-based analysis, 59.5% of patients had distant metastases at diagnosis [34]. Reported lymph node metastasis rates range from 50.4% to 84.6%, while liver metastasis is the most frequent distant event and occurs in 41.9% to 75.6% of gastric cases [34,39]. Even after curative-intent surgery, recurrence remains common. In a resected HAS cohort, the recurrence rate was 44%, the liver was the first site of relapse in 62.2% of cases, and the median time to recurrence was only 9 months [40,41]. Thus, surgery alone is rarely sufficient for cure [34,40]. Historically, conventional cisplatin/5-FU-based therapy produced poor outcomes in advanced disease, with median OS only 5–11 months [33,34].

More recently, a multicenter study of 25 patients with advanced HAC treated with PD-1 blockade plus chemotherapy as first-line therapy showed that the objective response rate was 76.0%, the disease control rate was 88.0%, median PFS was 10.2 months, and median OS reached 20.3 months [42]. Similarly, camrelizumab plus apatinib and SOX produced an objective response rate of 66.7%, a disease control rate of 88.9%, median PFS of 7.8 months, and median OS of 18.0 months in a 36-patient cohort [43,131]. Across published analyses, 5-year OS is approximately 8–9%, while 3-year OS ranges from 16.9% to 22.6% in unselected cohorts; by contrast, resectable disease may achieve 3-year survival rates of 58.1–61.2% [34,40] (Table 1).

### 4.2. Adenocarcinoma with Enteroblastic Differentiation

Histologically, adenocarcinomas exhibiting enteroblastic differentiation display a primitive intestinal-type architecture, consisting of cuboidal to columnar epithelial cells with clear cytoplasm [45,46,47] (Figure 3C–F). By immunohistochemistry, tumor cells show variable expression of SALL4, claudin-6, and Glypican-3 [48]. Recent literature proposes that gastric adenocarcinoma with enteroblastic differentiation can be diagnosed if at least one of the three markers (AFP, Glypican-3, or SALL4) is positive [49]. Kraemer et al. observed a consistent loss or marked reduction in cytokeratin 7 (CK7) expression in this subgroup, distinguishing it from conventional adenocarcinoma [48]. The authors further acknowledged a methodological limitation of tissue-microarray (TMA) screening, in which intratumoral heterogeneity may obscure positive cases; therefore, a stringent cutoff of ≥ 50% marker positivity was applied to ensure biological relevance [48]. Notably, claudin-6 expression, which is typically detected in >90% of testicular cancers, is only seen in ~3% of gastric subtypes [48]. Kraemer et al. observed a loss of SMARCA2 expression, while one patient additionally exhibited loss of ARID1A [48]. Both proteins are subunits of the SWI/SNF chromatin-remodeling complex, which is disrupted in up to 25% of all human malignancies [50]. Limited evidence also suggests involvement of other DNA damage–related pathways in this subtype. In a Japanese case series, an *ATM* mutation was identified in only one of 51 cases, whereas *TP53* promoter methylation and loss of heterozygosity at the *TP53* locus were detected in 18% and 37.2% of tumors, respectively [132]. More recently, whole-exome sequencing of an individual case identified pathogenic mutations in *TP53*, *KLHL7*, *RAPSN*, and *ACTA1* [133]. Although the oncogenic consequences of these alterations remain to be fully elucidated, current hypotheses implicate genomic instability, defective DNA-repair pathways, lineage-specific epigenetic remodeling, and activation of unique oncogenic signaling networks [134,135,136,137,138,139]. Although exceedingly rare, analogous to ROS1-altered non-small-cell lung cancer (NSCLC) [140], such small molecularly defined sub-entities can have major therapeutic implications. These molecular findings bear translational relevance, as multiple early-phase clinical trials are exploring poly(ADP-ribose) polymerase (PARP) inhibitors and Enhancer-of-Zeste-Homolog-2 (EZH2) inhibitors to therapeutically target SWI/SNF-deficient tumors [50].

Clinically, adenocarcinomas with enteroblastic differentiation appear to pursue a more aggressive course than conventional adenocarcinomas, with a high propensity for early metastasis, particularly in cases associated with elevated serum AFP levels. There is no established standard treatment for metastatic disease. Reported localized cases have generally been managed with cisplatin plus 5-fluorouracil (CF) as the most commonly employed first-line adjuvant chemotherapy regimen. However, the efficacy of this approach remains controversial, and outcomes have been poor despite multimodal treatment, including surgery and chemoradiotherapy. The role of immune checkpoint inhibitors remains undefined and empirical, and may be limited by immune evasion mechanisms, which currently serves as the primary biological reference given the paucity of data, including HLA-G expression and HLA class I deficiency [141]. A Chimeric Antigen Receptor T-cell (CAR-T) therapy directed against claudin-6 is presently being evaluated in a clinical trial by BioNTech (BNT-211-01), enrolling patients with claudin-6 expression in >50% of tumor cells and refractory to approved treatment lines [51]. Beyond claudin-6, Glypican-3 (GPC3) has also attracted attention as a candidate target for antibody-based and CAR-T therapies in solid tumors [51]. GPC3-directed immunotherapies are predominantly investigated in hepatocellular carcinoma [142] and no active clinical trials currently address GPC3-targeting in gastric or esophageal adenocarcinomas [143] (Table 1).

## 5. Adenosquamous Carcinoma

Adenosquamous carcinoma (ASC) is characterized by the coexistence of both glandular (adenocarcinoma) and squamous (SCC) malignant components, which may appear intermingled or arranged in parallel patterns within the same lesion [52] (Figure 2C–F).

ASC represents one of the rarest histological variants of esophageal and gastric malignancies, accounting for less than 1% of all primary esophageal and gastric carcinomas [53]. The largest reported cohort to date, described by Chen and colleagues in 2013, included only thirty-seven cases [54]. The typical age of onset for ASC is around the sixth decade of life [55], with a marked male predominance. Most tumors arise in the mid-esophagus, and both their clinical manifestations and macroscopic appearance are nearly indistinguishable from conventional SCC [55]. Because of this overlap and tissue sampling bias (i.e., only sampling the adenocarcinoma component and not the SCC component, or vice versa), ASC is often misdiagnosed preoperatively. According to the guidelines for clinical and pathological studies of carcinomas of the esophagus established by the Japanese Society for Esophageal Diseases, diagnosis requires that the minor component, either adenocarcinoma or SCC, constitutes at least 20% of the overall tumor mass. According to WHO classification, however, ASC is defined by the presence of a substantial SCC component interwoven with tubular adenocarcinoma elements, without specifying any particular proportion between the two histologic components [56]. Dedicated epidemiologic studies defining risk factors specific to adenosquamous carcinoma of the esophagus or stomach are lacking due to its rarity. Accordingly, risk factors are generally presumed to overlap with those established for conventional ESCC and/or esophageal adenocarcinoma.

The origin and pathogenesis of ASC remain subjects of ongoing debate. One theory suggests that the tumor develops from a single neoplastic clone capable of differentiating into both squamous and glandular lineages. Another hypothesis proposes that ASC arises from a pre-existing squamous carcinoma in which a subset of tumor cells later undergoes glandular metaplasia [144]. The esophageal environment may also play a role in this transformation. Since the esophagus maintains a higher pH than the stomach, squamous cells that migrate or undergo metaplastic changes within a more acidic environment, such as at the GEJ, may not survive. This cellular vulnerability could explain the rarity of identifiable squamous components in gastric or lower esophageal lesions and the overall low incidence of ASC [54,145].

To date, no studies have specifically applied RNA sequencing, whole-exome sequencing, or integrated genomic–transcriptomic analyses to gastroesophageal adenosquamous carcinoma as a distinct histologic entity. Consequently, the molecular drivers, transcriptional programs, and genomic alterations underlying the dual squamous and glandular differentiation of ASC are largely inferred from conventional adenocarcinoma and SCC datasets. From a biological perspective, ASC demonstrates a more aggressive behavior than either pure adenocarcinoma or SCC. It tends to metastasize to regional lymph nodes early in the disease course [53]. The adenocarcinoma component appears to play a key role in shaping the biological behavior of gastric adenosquamous carcinoma (GASC) [57,146]. The median survival ranged from 12 to 24 months, although outcomes vary substantially by tumor site, stage, and resectability [53]. Complete surgical resection remains the cornerstone of management when clinically possible [58,59]. Recent research has shown that patients with primary GASC more frequently demonstrate PD-L1 positivity and deficient mismatch repair (dMMR) status, indicating that immunotherapy may serve as a viable first-line systemic treatment option for these cases [60].

In summary, adenosquamous carcinoma represents a rare but biologically aggressive malignancy of the esophagus and stomach, characterized by mixed glandular and squamous differentiation. Given the limited molecular data available, future genomic and transcriptomic studies are needed to better identify potential therapeutic vulnerabilities (Table 1).

## 6. Neuroendocrine Carcinoma

**Figure 4:**
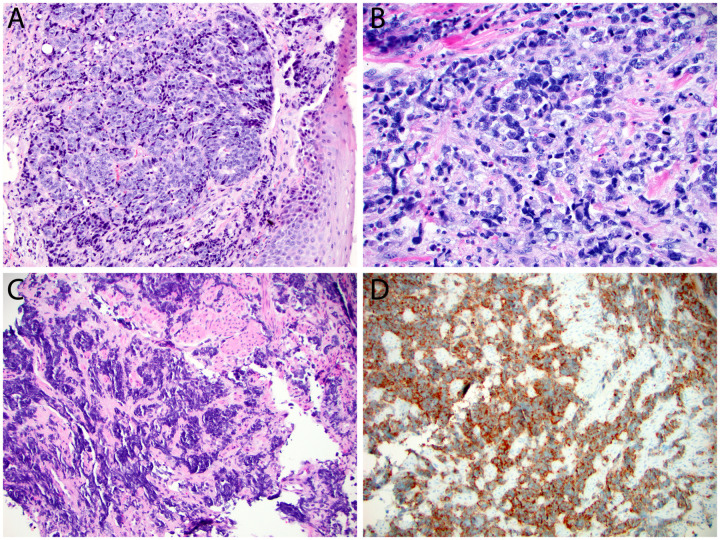
Representative photographs of poorly differentiated neuroendocrine carcinoma. (**A**) Large cell neuroendocrine carcinoma present beneath squamous epithelium (H&E, original photo at 100× mag.) (**B**) High magnification view of esophageal large cell neuroendocrine carcinoma showing nests and sheets of tumor cells with moderate amounts of cytoplasm and pleomorphic nuclei with prominent nucleoli. Mitoses and single cell necrosis are frequent (H&E, original photo at 400× mag.). (**C**) Esophageal small cell neuroendocrine carcinoma showing compact cells with scant cytoplasm, nuclear hyperchromasia, and nuclear molding and crush artefact (H&E, original photo at 200× mag.). (**D**) Synaptophysin immunostain of esophageal small cell neuroendocrine carcinoma, showing diffuse reactivity (synaptophysin IHC, original photo at 200× mag.). Legend: IHC, immunohistochemistry; mag., magnification.

A neuroendocrine neoplasm (NEN) is a tumor derived from or showing differentiation toward neuroendocrine cells [61]. These cells are characterized, in part, by expression of neuroendocrine markers, including chromogranin A, neuron-specific enolase (NSE), synaptophysin, and INSM1 [61]. Epithelial NENs are broadly divided into two major categories: well-differentiated neuroendocrine tumor (WDNET) and poorly differentiated neuroendocrine carcinoma (PDNEC) [61]. WDNETs are composed of uniform well-differentiated tumor cells with finely granular cytoplasm and moderate amounts of cytoplasm and stratified into grades G1, G2, or G3 using mitotic count and the Ki-67 proliferation index; discussion of WDNETs is beyond the scope of this review. PDNECs are characterized as high-grade, poorly differentiated carcinomas that encompass both small-cell neuroendocrine carcinomas (SCNECs) and large-cell neuroendocrine carcinomas (LCNECs). SCNECs are characterized by compact cells with hyperchromatic nuclei, scant cytoplasm, and nuclear molding, whereas LCNECs contain cells with pleomorphic nuclei, prominent nucleoli, and moderate to abundant cytoplasm (Figure 4). Furthermore, a small subset of SCNECs and LCNECs may be admixed with a non-neuroendocrine carcinoma, typically either SCC or adenocarcinoma. These so-called mixed neuroendocrine-non neuroendocrine neoplasms are defined by the presence of at least 30% of each component in the tumor [147]. Sequencing studies indicate that the neuroendocrine and non-neuroendocrine components of these lesions have a common clonal origin [148].

Esophageal neuroendocrine neoplasms (E-NENs) are exceedingly uncommon, representing approximately 0.03% of all esophageal malignancies [62]. Esophageal neuroendocrine carcinoma (E-NEC) accounts for roughly 6–56% of primary gastrointestinal NECs [149]. E-NEC comprises about 0.3–1.0% of all esophageal cancers and occurs more frequently in Asian populations compared with Western cohorts [62]. E-NEC occurs predominantly in men, most often between the fifth and seventh decades of life. No definitive predisposing risk factors have been suggested to date. The mid-esophagus represents the most common primary location [150,151]. Dysphagia represents the most common symptom, while patients with more advanced disease may experience esophageal obstruction [152,153]. The tumor typically appears as a single, large, fungating lesion, though multifocal occurrences have occasionally been described [154].

Clinically, the disease course does not correlate with the degree of immunoreactivity for endocrine markers [152,153]. Distant metastases most commonly involve the liver, lungs, and bones, while brain metastases are relatively uncommon [150,151]. Epidemiological data from large registries have shown a five-year OS of only about 20% for PDNEC [155]. Overall, it appears to carry a substantially worse prognosis than conventional ESCC or adenocarcinoma, largely due to early systemic dissemination. Liquid biopsy approaches such as NETest have emerged as promising tools for monitoring tumor progression and predicting therapeutic response in NENs, where high baseline NETest levels correlate with disease advancement [156].

In a large retrospective study analyzing 147 SCNEC cases, no PD-L1 expression was detected in tumor cells, a pattern consistent with SCLC and other extrapulmonary small cell carcinomas [63,157,158,159]. In contrast, PD-L1 expression in TIICs was observed in nearly half of the cases [63,157,160], with a mean CD8^+^ TIL density of 195.9 cells/mm^2^, comparable to that observed in SCLC [63]. Genomic analyses by Wang et al. further demonstrated molecular similarities between SCNEC and SCLC, supporting shared biological features between these entities [161]. To date, comprehensive RNA sequencing–based transcriptomic analyses specific to SCNEC have not been systematically reported, and most molecular insights are derived from DNA-level analyses and extrapolation from SCLC. Both PD-L1 expression in TIICs and CD8^+^ TIL density were significantly associated with improved PFS and OS in SCNEC [63,160]. PD-L1 expression quantified by CPS was recognized as a prognostic factor for OS [64,65,160], while CD8^+^ TIL density independently predicted relapse-free survival and OS [63], consistent with findings reported in SCLC [162,163].

Based on PD-L1 expression and CD8^+^ TIL density, SCNEC tumors were classified into four immune phenotypes: Type I (PD-L1^+^/CD8^+^), Type II (PD-L1^−^/CD8^−^), Type III (PD-L1^+^/CD8^−^), and Type IV (PD-L1^−^/CD8^+^) [63,160]. Among these, Type II tumors exhibited the poorest survival outcomes [63,160]. Collectively, these findings indicate that PD-1/PD-L1–targeted immunotherapy may preferentially benefit SCNEC patients with a Type I immune phenotype characterized by pre-existing activated T-cell infiltration [157].

The U.S. Food and Drug Administration (FDA) has approved PD-L1 inhibitors, atezolizumab and durvalumab, as first-line therapy, and nivolumab and pembrolizumab, as third-line monotherapies for small cell lung cancer (SCLC) [157]. However, the clinical efficacy of PD-1/PD-L1 inhibitors in small cell neuroendocrine carcinoma (SCNEC) of the esophagus remains insufficiently characterized [164]. Across multiple cancer types, PD-L1 expression on tumor cells (TCs) or tumor-infiltrating immune cells (TIICs), as well as the density of CD8^+^ tumor-infiltrating lymphocytes (TILs), have been associated with improved outcomes following immune checkpoint inhibitor (ICI) therapy [165,166,167,168], although their prognostic and predictive significance in SCNEC has not been well defined [169,170,171].

Overall treatment for E-NECs should be based on a platinum-containing doublet chemotherapy regimen [172]. Surgical resection may be considered for patients in whom the tumor is technically resectable. The National Comprehensive Cancer Network (NCCN) guidelines recommend a combination of chemotherapy and radiotherapy, although a universally accepted standard of care for patients with locally advanced E-NEC has not yet been established. In a study conducted by Kikuchi and colleagues, patients with clinical stage I–III E-NEC showed different survival outcomes depending on the treatment strategy: the OS was significantly longer in both the adjuvant and neoadjuvant chemotherapy groups compared with surgery alone [66]. However, no difference in OS was observed between adjuvant and neoadjuvant chemotherapy, or between chemotherapy with or without radiotherapy and neoadjuvant approaches. Neoadjuvant chemotherapy may therefore be a reasonable option for patients with resectable, locally advanced E-NEC [67].

Definitive chemoradiotherapy has also demonstrated encouraging outcomes in patients with locally advanced E-NEC [68]. Honma et al. in Japan evaluated the efficacy and safety of this approach, which consisted of radiotherapy delivered at 60 Gy in 30 fractions, combined either with platinum plus etoposide or with cisplatin and 5-FU. The overall response rate reached 86.4%, with a clinical complete remission rate of 77.3%. The median PFS was 12.7 months, and the median OS was 37.5 months [69].

Patients with metastatic or recurrent E-NEC are generally treated with palliative chemotherapy. According to the 2019 Japanese guidelines for GEP-NEN, recommended regimens consist of a platinum-based agent in combination with either etoposide or irinotecan [67]. The NCCN guidelines propose several potential first-line options, including etoposide plus cisplatin (EP), etoposide plus carboplatin (EC), irinotecan plus cisplatin (IP), irinotecan plus carboplatin, as well as FOLFOX, FOLFIRI, FOLFIRINOX, or the combination of temozolomide and capecitabine (CAPTEM). In the JCOG1213 (TOPIC-NEC) phase III trial, which included patients with E-NEC, no difference in OS or PFS was found between the etoposide–cisplatin (EP) and irinotecan–cisplatin (IP) regimens [70].

EC therapy, consisting of etoposide combined with carboplatin, is considered an appropriate alternative for patients with metastatic or recurrent NEC who are unable to tolerate cisplatin [173]. Sorbye and colleagues suggested a response rate of 30% and a median OS of 11 months, results that were similar to those observed with the EP regimen [173]. Given that topotecan or irinotecan-based regimens are well-established second-line treatments in SCLC, irinotecan-containing chemotherapy may represent a rational subsequent option in E-NEC. In this context, emerging evidence suggests that the FOLFIRI regimen (5-fluorouracil, leucovorin, and irinotecan) [174], as well as the FOLFOX regimen (5-fluorouracil, leucovorin, and oxaliplatin) [175], may offer potential clinical benefit following failure of first-line chemotherapy.

In summary, E-NEC is a rare but highly aggressive malignancy with distinct biologic and immunologic features, limited molecular characterization, and a persistently poor prognosis (Table 1).

## 7. Adenoid Cystic Carcinoma

Esophageal adenoid cystic carcinoma (EACC), accounting for approximately 0.1% of all cases, and is believed to originate from myoepithelial cells and the intercalated ducts of the submucosal esophageal glands [71]. In earlier reports, some cases diagnosed as esophageal adenoid cystic carcinoma may in fact represent basaloid squamous cell carcinoma (BSCC), which in some cases can show morphologic overlap with EACC but has distinct clinicopathologic features and a different clinical course. Specifically, ACC occurs more frequently in women and appears to have a more favorable prognosis [176]. Current evidence suggests that lifestyle and anatomical factors such as smoking, alcohol consumption, obesity, and the presence of a hiatal hernia may play an important role in the pathogenesis of EACC [71].

**Figure 5:**
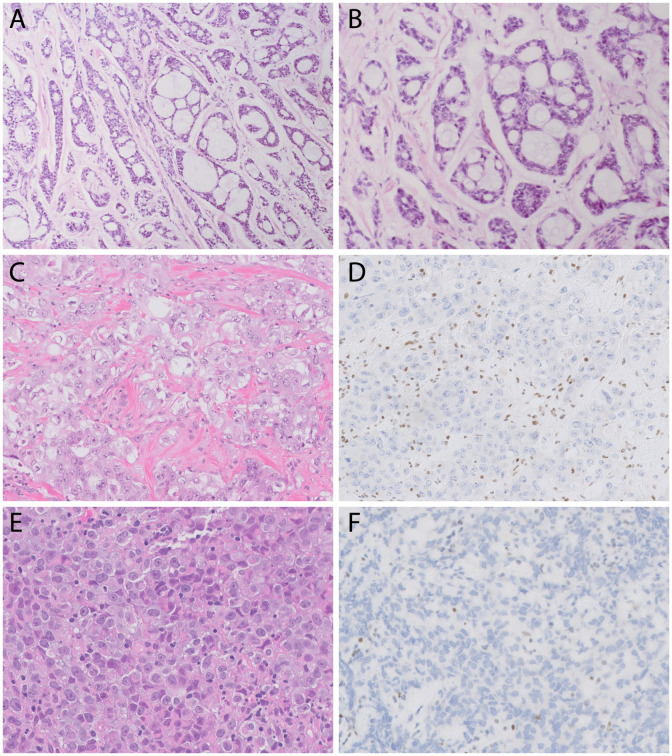
Representative photographs of adenoid cystic carcinoma (**A**,**B**) and SWI/SNF-complex deficient tumors (**C**–**F**). (**A**) An example of adenoid cystic carcinoma, showing infiltrating nests and cords of tumor cells with cribriform gland arrangements (center) (H&E, original photo at 100× mag.). (**B**) High magnification view of adenoid cystic carcinoma, showing a mixed population of glandular/ductal cells with eosinophilic cytoplasm, and compact basal/myoepithelial cells (H&E, original photo at 200× mag.). (**C**) SMARCB1-deficient tumor (H&E, original photo at 200× mag.). (**D**) Tumor shows loss of INI-1 expression with background admixed inflammatory cells showing retained expression (INI-1 IHC; original photo at 200× mag.). (**E**) SMARCA4-deficient tumor (H&E, original photo at 400× mag.). (**F**) Tumor shows loss of BRG1 expression with background admixed inflammatory cells showing retained expression (BRG1 IHC; original photo at 400× mag.). Legend: IHC, immunohistochemistry; mag., magnification.

The tumor most frequently occurs in the middle third of the esophagus, followed by the lower and upper portions. Histologically, the neoplasm resembles tumors of a same name which arise in the salivary gland and lung (Figure 5A,B), with ductal and myoepithelial cells arranged in tubular, cribriform, and solid patterns. The tumor is infiltrative and perineural invasion is frequent. When compared with ACC arising in the head and neck region, EACC tends to display a more aggressive clinical course, with common sites of distant metastasis including the lungs and bones [177]. The most common endoscopic appearance of EACC is a bulging lesion (approximately 58.6%), while ulceration accounts for about a quarter of the cases [178]. In some instances, eosinophilic infiltration has been observed, raising the possibility of an association with eosinophilic esophagitis or chronic mucosal irritation [72].

The tumor is composed of both epithelial cells and myoepithelial cells variably arranged in cribriform, tubular/glandular, and solid architectures. Epithelial cells generally express cytokeratins, CEA, and CD117, whereas the myoepithelial component is positive for smooth muscle actin (SMA), S100, CK5/6, p63, p40, and calponin. Rearrangements involving *MYB*, *MYBL1*, and/or *NFIB* are seen as the genomic hallmark of ACC of salivary glands of the head and neck and tracheobronchial tree [179,180]. The defining genomic feature of the tumor is t(6;9) or t(8;9) translocation, leading to *MYB*::*NFIB* and *MYBL1*::*NFIB* fusions, respectively. *MYB::NFIB* fusions are identified in more than 50% of patients, whereas *MYBL1::NFIB* fusions are observed in about 5% [179,180]. Literature regarding genetic alterations in EACC is not as robust; however, the genetic underpinning of EACC is likely the same as ACC elsewhere.

The prognosis of EACC remains difficult to establish due to its rarity and heterogeneous behavior. Lymph node metastasis and vascular invasion have been identified as poor prognostic indicators. Endoscopic misdiagnosis is frequent, reported in nearly 78% of cases, most often confused with ESCC or esophageal leiomyoma [181]. Some reports describe EACC as a highly aggressive malignancy with frequent systemic dissemination, yielding a one-year survival rate of 23% and a median OS of approximately seven months [73]. In contrast, Dutta et al. reported a more favorable five-year survival rate of 47% [182]. Hiromichi et al. observed that patients with EACC whose tumors lacked any SCC or basal cell carcinoma (BSC) components had an average OS of approximately 25 months [183].

When possible surgical resection remains the cornerstone of therapy and is considered the most effective approach for localized disease [71,74,182]. Nonetheless, various treatment options have been explored. A previous report described combination chemotherapy using doxorubicin, mitomycin C, and 5-FU as a potentially effective regimen in EACC [74]. However, most evidence suggests that chemotherapy alone may not provide meaningful benefit [184]. Interestingly, Yoshikawa et al. reported the first case of EACC treated successfully with endoscopic submucosal dissection [185]. Some success has been reported with the combination of cetuximab and radiotherapy, as documented by Jensen et al., but the limited number of cases prevents firm conclusions regarding efficacy [75] (Table 1).

## 8. Undifferentiated Carcinoma

Undifferentiated carcinoma of the esophagus and stomach is one of the most aggressive and diagnostically challenging malignancies of the upper gastrointestinal tract. In the reviewed literature, these tumors are defined by the absence of overt squamous, glandular, or neuroendocrine differentiation and are increasingly understood as epigenetically driven neoplasms linked to disruption of the SWI/SNF chromatin-remodeling complex [76,77]. Although epidemiologic estimates remain limited, available series indicate a marked male predominance, a broad age range of 39–84 years, and a tendency to the lower part of the esophagus or GEJ, often in association with Barrett’s esophagus [76,78,79]. Clinically, patients usually present with dysphagia, profound weight loss, chest or epigastric pain, or bleeding, but the tempo of disease progression appears more explosive than in routine adenocarcinoma or SCC [76,80,81].

Undifferentiated carcinoma typically shows sheets or discohesive clusters of pleomorphic cells with vesicular nuclei, brisk mitotic activity, extensive geographic necrosis, and, in many cases, rhabdoid cytomorphology [76,77] (Figure 5C–F). Because lineage-specific differentiation is absent, the main practical pitfall is misclassification as poorly differentiated neuroendocrine carcinoma. This problem is compounded by frequent synaptophysin positivity. In one study of SMARCA4-deficient esophageal undifferentiated carcinoma, 8 of 22 cases were initially interpreted as neuroendocrine carcinoma because of synaptophysin expression [79]. For this reason, diagnosis should not rely on a single neuroendocrine marker. A more informative panel includes keratin AE1/AE3, synaptophysin, chromogranin A, BRG1 (SMARCA4), BRM (SMARCA2), INI1 (SMARCB1), and, in select esophageal cases, SALL4. Chromogranin negativity, focal or weak cytokeratin expression, and complete loss of any SWI/SNF complex protein favor undifferentiated carcinoma over true neuroendocrine carcinoma [76,77]. This distinction is clinically important because therapeutic extrapolation from NEC can be misleading.

The most important biologic advance in this area has been recognition of SWI/SNF deficiency, especially SMARCA4 loss. In a cohort of 1174 esophageal and gastric carcinomas, *SMARCA4* alterations were identified in 9.1% of cases, but only 3.6% represented pathogenic variants; complete loss of SMARCA4 expression by immunohistochemistry was reported in 2.0% of 1199 tumors [78,82]. Among pathogenic variants, 71% occurred in the esophagus or GEJ and 29% in the stomach, and truncating variants were much more strongly associated with undifferentiated morphology than missense variants (64% vs. 25%). Additional co-alterations included *APC* in 31%, *CTNNB1* in 14%, *TP53* in 76%, and *ARID1A* in 31% [78]. These findings support the concept that undifferentiated carcinoma is not merely a poorly differentiated epithelial tumor, but a biologically distinct subtype in which epigenetic deregulation drives dedifferentiation, invasive growth, and treatment resistance.

Current treatment outcomes remain poor [76]. Both lymphatic and hematogenous dissemination are common, with liver, lung, and bone representing the dominant metastatic sites; in the liver-metastatic cases summarized by Xu et al., death occurred within 72–78 days of diagnosis [83]. Conventional chemotherapy has generally been disappointing. In comparative data cited in the reviewed literature, differentiated adenocarcinoma achieved a 68% objective response rate to UFT-EAP, whereas undifferentiated carcinoma achieved only 20%, with a median survival of 10 months and a median duration of response of 12.2 months in responders [186]. Some early signals suggest biomarker directed approaches may improve outcomes, including immunotherapy in metastatic microsatellite stable *ARID1A*-mutant esophageal undifferentiated carcinoma achieving a durable response to pembrolizumab for more than 2 years [84]. In another reported case of SMARCA4-deficient gastric undifferentiated carcinoma, treatment with cadonilimab plus anlotinib produced 33 months of recurrence-free survival [85]. SWI/SNF-driven epigenetic immune escape may blunt the benefit of immune checkpoint inhibitors despite apparently favorable immune biomarkers like PD-L1 and tumor sequencing may be more clinically important in this subtype [86]. Routine BRG1/SMARCA4 testing should be considered in all poorly differentiated esophageal and gastric carcinomas, particularly when synaptophysin is positive and chromogranin is negative [77,79]. Beyond diagnosis, SWI/SNF deficiency opens a therapeutic framework in which EZH2 inhibition represents a synthetic-lethal strategy, etoposide may serve as a non-FLOT alternative in selected resistant cases, and combined epigenetic therapy with PD-1 blockade remains investigational but biologically compelling [85,86,87] (Table 1).

## 9. Gastrointestinal Stromal Tumor

Gastrointestinal stromal tumor (GIST) represents the most prevalent mesenchymal neoplasm of the gastrointestinal (GI) tract, yet it represents only about 2% of all gastrointestinal tumors. Many small gastric GISTs are detected incidentally during endoscopy, imaging, or abdominal surgery performed for unrelated conditions. While the stomach is the primary site of origin, these lesions can also arise, though far less commonly, in the esophagus. In symptomatic esophageal GIST, dysphagia is the most common presenting symptom, whereas gastrointestinal bleeding, more frequently observed in gastric GISTs, occurs in only about 10% of esophageal GIST [187,188]. Anatomic location, tumor size, and mitotic rates are important components of risk stratification in GIST. Esophageal GIST have been suggested to carry a poorer prognosis than gastric GIST; however, most available evidence is derived from small case series [189]. Patients with larger tumors (>2 cm) and tumors with higher mitotic rates (i.e., >5 mitotes per 5 mm^2^) are more at risk for progressive disease [188].

**Figure 6:**
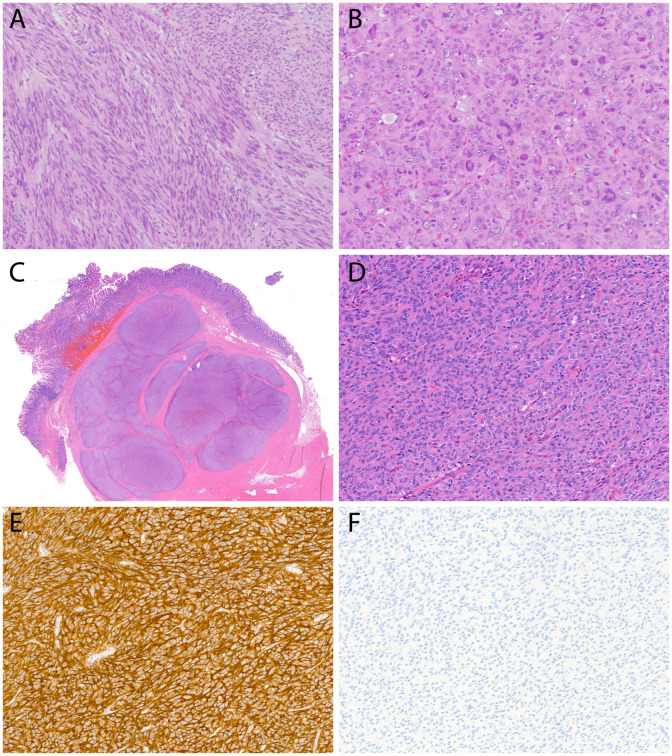
Representative photographs of gastrointestinal stromal tumor (GIST). (**A**) GIST with spindle cell morphology without conspicuous mitotic activity. Tumor was positive for DOG1. (H&E, original photo at 200× mag.). (**B**) GIST with epithelioid morphology, pleomorphism, and high mitotic activity. Other sections of the tumor show prominent necrosis. Tumor was positive for DOG1 (H&E, original photo at 200× mag.). (**C**–**F**). SDH-B deficient GIST. (**C**) Lower power image demonstrates multilobular architecture characteristic of the lesion (H&E, original photo at 10× mag.). (**D**) Higher power image shows epithelioid morphology without conspicuous mitotic activity (H&E, original photo at 200× mag.). (**E**) DOG1 immunostain is diffusely positive (DOG1 IHC, original photo at 200× mag.). (**F**) SDH-B immunostain shows complete absence of staining (SDH-B IHC, original photo at 200× mag.). Legend: IHC, immunohistochemistry; mag., magnification.

Approximately 70% of GIST show spindle cell morphology, whereas around 20% display epithelioid morphology, with the remainder exhibiting mixed phenotypes [88] (Figure 6). This wide histologic variability poses diagnostic challenges when assessment is based solely on morphologic characteristics [89]. The histologic differential frequently includes smooth muscle and nerve sheath tumors (in cases with spindle cell morphology) along with potentially melanoma, neuroendocrine tumors, and carcinomas (in cases with epithelioid morphology). A select panel of immunostains is frequently helpful; tumor positivity for CD117 (cKIT) and DOG1 (further discussed later) is supportive of a diagnosis of GIST while positivity for desmin and/or S100 is less typical for GISTs and suggests an alternative diagnosis. We would direct readers to the following excellent GIST reviews [88,187,188]. The diagnostic approaches, treatment paradigms, and prognosis have been reviewed in detail and briefly summarized below for completeness.

In the management of localized GIST, complete surgical resection continues to be the primary treatment approach. However, surgical management of esophageal GIST is technically more challenging because of the proximity to critical mediastinal structures. As a result, tumor enucleation has been considered in selected cases to avoid formal esophageal resection, which is associated with substantial morbidity. Despite complete resection, long-term surveillance remains essential because the risk of recurrence is determined by tumor size, mitotic activity, anatomic location, and tumor rupture, and recurrences may occur years after surgery. In patients with technically unresectable tumors or those at high risk for surgical complications neoadjuvant therapy with tyrosine kinase inhibitors has been increasingly explored; however, the supporting evidence is more robust for gastric GIST [190,191].

The medical therapeutic landscape of GIST changed markedly following the recognition that activating mutations of *KIT* drive tumorigenesis in a large proportion (about 75%) of cases [88,90]. However, not all GIST have *KIT* mutations; a subset of these tumors (approximately 10%) harbor activating platelet-derived growth factor receptor alpha (*PDGFRA*) mutations [188]. For these lesions, immunohistochemical staining for DOG1, a transmembrane protein overexpressed in GIST, has proven to be a more reliable diagnostic marker than CD117 [91]. DOG1 expression is not entirely specific to GISTs and has been rarely reported in other mesenchymal neoplasms and carcinomas [91].

Alterations involving *KIT* exon 11 represent the most frequently identified molecular abnormalities in GIST. Compared with *KIT* exon 11-mutated tumors, GIST harboring *KIT* exon 9 mutations generally demonstrate a more aggressive clinical behavior and less favorable disease course [192]. The discovery of activating mutations of KIT driving tumorigenesis enabled the development of tyrosine kinase inhibitors as effective targeted therapies [193]. GIST with *KIT* exon 11 mutations are typically more responsive to imatinib, an orally administered tyrosine kinase inhibitor and frequently achieve more durable responses, whereas tumors with *KIT* exon 9 mutations tend to exhibit reduced sensitivity [92]. In addition, certain mutations, particularly *PDGFRA* exon 18 D842V, are considered intrinsically resistant to imatinib [92,194,195,196].

Further molecular analyses have identified an additional small subset of GIST that lack mutations in both *KIT* and *PDGFRA*. These tumors show alterations in *SDH* subunit genes, *NF1*, *BRAF,* or *KRAS* [93,197]. SDH-deficient GISTs are generally considered relatively indolent with an overall low mortality rate, despite frequent disease progression and tumor recurrence [198]. Patients with these tumors frequently have mutations in the *SDHB* gene as part of the Carney triad or Carney–Stratakis syndrome, characterized by GIST, paragangliomas, and pulmonary chondromas [198]. Beyond genetic mutations, several chromosomal abnormalities, most notably loss of heterozygosity (LOH) at 1p, 14q, and 22q, have been reported [199].

Collectively, GIST represent a molecularly defined mesenchymal neoplasm in which integrated morphologic, immunophenotypic, and genomic assessment is essential for accurate diagnosis, prognostication, and therapeutic decision-making (Table 1).

## 10. Gastroblastoma

Gastroblastoma is an ultra-rare biphasic gastric neoplasm first described in 2009 [200]. Approximately 27 cases have been reported worldwide [99]. Although initially considered a tumor of children, later reports expanded the range to 74 years with a mean of 35 and no clear sex predominance [99]. The antrum is the most common site, although body, greater curvature, and pyloric lesions extending into the duodenum have also been described [200,201]. Tumors have been reported to range in size from 1.3 to 15 cm [99]. Clinically, patients usually present with epigastric pain [100], melena [202], chronic iron deficiency anemia [202], and occasionally gastric outlet obstruction [201]; asymptomatic incidental detection has also been reported [203]. In contrast to routine gastric carcinomas, many gastroblastomas appear as submucosal-type lesions and are therefore mistaken preoperatively as GIST [99].

Endoscopically, gastroblastoma often appears as a submucosal mass covered by intact mucosa [100]. EUS usually demonstrates a heterogeneous hypoechoic lesion arising from the fourth layer, the muscularis propria [202], and computed tomography may show a well-circumscribed enhancing mass, occasionally with small cystic foci; however, none of these findings is pathognomonic [99]. Histologically, the tumor shows both epithelial and mesenchymal elements. The epithelial portion can appear primitive with variable amounts of islands, tubules, or rosette-like structures. The mesenchymal portion is frequently uniform with only mildly atypical spindled cells [100,200]. By immunohistochemistry, the epithelial component is positive for keratin AE1/AE3, CAM5.2, and CK7 while the mesenchymal component is positive for GLI1, vimentin, CD10, and CD56 [100]. By contrast, CD117, DOG1, SMA, desmin, S100, and SOX10 are generally negative, which is diagnostically useful in excluding GIST and other spindle cell neoplasms [100]. At the molecular level, recurrent GLI1-pathway rearrangements provide additional specificity [100]. *MALAT1*::*GLI1* appears to be the most frequent fusion [100], while *ACTB*::*GLI1* [204], *PTCH1*::*GLI2* [203], and *EWSR1*::*CTBP1* [99] have also been reported. Accordingly, next-generation sequencing [204] or break-apart FISH [99] may be particularly valuable in diagnostically difficult cases. Although most reported cases occur in children and young adults, adult cases appear to share the same characteristic biphasic morphology. However, given the extremely limited number of reported cases, whether age-related biological differences exist remains unknown.

Complete surgical excision with negative margins remains the therapeutic keystone [99]. In pooled analyses of 27 reported cases, 23 patients (85.2%) underwent gastrectomy in the form of partial, subtotal, or total resection, whereas 3 patients underwent endoscopic resection procedures such as ESD, ESE, or EFTR, and 1 patient underwent laparoscopic-endoscopic cooperative surgery [99].Tumor size appears to influence treatment selection: the mean tumor size was 1.91 cm in patients managed endoscopically compared with 5.80 cm in those undergoing open or laparoscopic surgery [99]. These data suggest that minimally invasive, organ-preserving strategies may be reasonable in carefully selected localized lesions [99]. Available case reports suggest generally favorable outcomes after complete surgical resection, with several patients remaining disease-free for 12–24 months [201,202,203]. Margin status appears central to local control [99]. Inadequate resection margins have been associated with early locoregional recurrence requiring further surgery [99].

The role of lymph node dissection remains unsettled, but available data argue against viewing gastroblastoma as uniformly low grade [99]. Lymph node metastasis has been documented in 3 of 27 patients (11.1%) [99,205]. Overall, 4 of 27 patients (14.8%) developed metastases, and 1 of 27 died of disease, corresponding to a disease-specific mortality of 3.7% [99]. Prognosis after complete resection is generally favorable [99,100]. Pooled outcome estimates suggest an OS of 96.3% and recurrence-free survival of approximately 85% [99]. The original series documented disease-free survival of 3.5, 5, and 14 years [201], and later case reports described recurrence-free follow-up of 9, 12, 19, 24, and 100 months [99]. Proposed adverse prognostic features include larger tumor size, particularly >5 cm, positive margins, nodal involvement, transmural invasion, and possibly higher mitotic activity [99].

No standard adjuvant systemic therapy has been established [99]. More than 90% of reported patients received no treatment beyond surgery and chemotherapy data is limited to case reports [99,205]. From a translational perspective, recurrent activation of the Hedgehog/GLI1 axis provides a biologically plausible therapeutic hypothesis for unresectable or metastatic disease [100]. Similarly, focal PD-L1 expression and HDAC2 expression have been described in isolated cases, raising the possibility of future exploration of immune checkpoint blockade or epigenetic therapy [100]. At present, however, these strategies remain hypothesis-generating rather than evidence-based [99] (Table 1).

## 11. Conclusions

**Figure 7:**
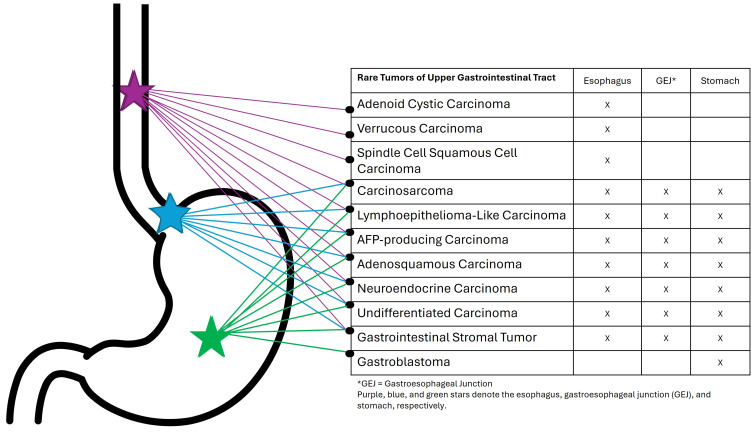
Site-specific distribution of rare esophageal, gastric, and gastroesophageal junction tumor subtypes.

Rare histologic subtypes of gastroesophageal malignancies represent a small but biologically meaningful fraction of upper gastrointestinal cancers (Figure 7). Although traditionally classified based on histopathologic appearance, the collective evidence reviewed here underscores that morphology alone is increasingly insufficient to capture the clinical behavior, prognosis, and therapeutic vulnerabilities of these tumors. Many of these rare subtypes share overlapping histologic features with more common carcinomas, frequently leading to misdiagnosis or delayed recognition, while their clinical outcomes are often driven by distinct molecular, immunologic, or lineage-specific characteristics rather than histology per se.

Accurate diagnosis remains the cornerstone of managing these rare malignancies. Given their overlapping morphologic features with more common gastroesophageal cancers, timely recognition requires integration of clinical presentation, high-quality imaging, histopathologic evaluation, immunohistochemistry, and, whenever appropriate, molecular profiling. Such a multidisciplinary diagnostic approach is essential for accurate classification, appropriate treatment selection, and optimal patient outcomes. Comprehensive genomic profiling, assessment of epigenetic regulators, immune microenvironment characterization, and identification of actionable biomarkers should be incorporated early into diagnostic workflows for rare esophageal and gastric tumors. Importantly, rare subtypes, despite their limited numbers, may harbor disproportionately informative biological insights and targetable vulnerabilities, offering opportunities for precision oncology approaches that extend beyond conventional treatment algorithms.

## Figures and Tables

**Table 1:** Summary of Clinicopathologic Features and Actionable Markers in Rare Gastroesophageal Tumors.

Clinical and Key Features	Molecular/Diagnostic Markers	Possible Therapeutic Targets
Esophageal Verrucous Squamous Cell Carcinoma [7,8,9,11,12,13,14,16]		
Extremely rare; <50–100 reported cases worldwide; mean age ~67 years (range 50–80); male predominance (2:1–3:1) Aggressive local behavior Refractory candidiasis/white plaque-like lesions; extensive superficial spread (2–22 cm); late diagnosis due to indolent course	Histology: Well-differentiated SCC variant with deceptively bland histology. Hyperkeratosis, verruciform (church-spire) architecture, pushing borders IHC: p16 negative, p53 wild-type pattern Molecular: HPV not implicated; frequent SMARCA4 alterations (>80%)	No established targeted or immunotherapy Potential sensitivity to chemoradiotherapy in selected cases (5-FU + cisplatin + radiotherapy) Management primarily local (surgery/endoscopic resection)
Spindle Cell Squamous Cell Carcinoma [17,18]		
~0.5–2.4% of esophageal neoplasms	Histology: biphasic morphology with both squamous and spindle cell component IHC: Squamous areas with cytokeratin-positivity; spindle areas with variable cytokeratin, vimentin, and SMA expression. Parallel p53 expression between both Reduced/absent E-cadherin in spindle transition	No validated therapeutic targets; biological pathways may include: p53 pathway dysregulation Epithelial–mesenchymal transition (EMT)-related alterations (E-cadherin loss) Mesenchymal signaling (vimentin/SMA) (biologically relevant but not clinically targetable)
Carcinosarcoma [19,20,21,22,23,24,25,26]		
~0.5–2.4% of esophageal malignancies Commonly arises in middle and lower esophagus	Histology: biphasic morphology with epithelial (carcinomatous) and mesenchymal (sarcomatous) components Shared genetic alterations between carcinomatous and sarcomatous elements supporting common clonal origin Evidence of epithelial–mesenchymal transition (EMT) and ZEB1 upregulation in sarcomatous component	Esophagectomy as primary curative approach Chemoradiation in selected cases; Adjuvant or multimodal therapy may be considered in advanced disease or high-risk patients (e.g., elevated preoperative neutrophil-to-lymphocyte ratio (NLR)) Prominent sarcomatous component associated with treatment resistance
Lymphoepithelioma-like Carcinoma [27,28,29,30,31,32]		
Esophageal cases extremely rare (39 reported cases by 2023) Mimics SCC clinically and histologically	Histology: overlap with SCC; Cytotoxic T-cell–rich infiltrate IHC: HMW keratins, p40, p63; High PD-L1 expression EBV association (rare in esophagus, frequent in gastric)	PD-1/PD-L1 axis EBV-associated immune pathways
Hepatoid Adenocarcinoma [33,34,35,36,37,38,39,40,41,42,43]		
Rare: incidence ~0.014–0.025 per 100,000; gastric type shows male predominance (~75.1%) Symptoms include epigastric pain, GI bleeding, jaundice depending on site	Histology: resembles hepatocellular carcinoma (HCC) IHC: HepPar-1, glypican-3, and arginase (all overlap with HCC); SALL4, BSEP, CDX2, and MDR3 (proposed specificity for hepatoid adenocarcinoma) Serum AFP elevated in majority but not all cases (≈52–87%)	PD-1 pathway (strong clinical signal); Anti-angiogenic therapy (e.g., apatinib) Intensified chemotherapy (triplet regimens in AFP-high disease) Potential multimodal approaches including surgery + systemic therapy
Adenocarcinoma with Enteroblastic Differentiation [44,45,46,47,48,49,50,51]		
E xtremely rare; <15 reported esophageal cases; ~0.7% in screening cohort	IHC: Variable expression of SALL4, Glypican-3, Claudin-6, and AFP—proposed diagnostic criterion fulfilled if ≥1 marker (AFP, GPC3, or SALL4) positive. Recurrent loss of SWI/SNF-complex subunits (SMARCA2, occasionally ARID1A)	Conventional chemotherapy (cisplatin + 5-FU) with limited efficacy Claudin-6–targeted CAR-T therapy under clinical investigation Potential targeting of SWI/SNF deficiency (e.g., PARP or EZH2 inhibition) Exploratory GPC3-directed immunotherapies
Adenosquamous Carcinoma [52,53,54,55,56,57,58,59,60]		
<1% of primary esophageal and gastric carcinomas (most commonly arises in mid-esophagus) Clinically and macroscopically resembles SCC Aggressive behavior with early lymph node metastasis (median survival 12–24 months)	Coexistence of malignant glandular and squamous components Higher frequency of CPS positivity and dMMR in gastric disease	Complete surgical resection Adjuvant chemoradiation in locally advanced disease Immunotherapy as potential first-line systemic treatment in CPS-positive and dMMR tums
Neuroendocrine Carcinoma [61,62,63,64,65,66,67,68,69,70]		
Extremely rare; 0.3–1.0% of esophageal cancers; usually arises in mid-esophagus Highly aggressive (median survival ~4–18 months)	Poorly differentiated carcinoma with small-cell (most frequent) or large-cell morphology Neuroendocrine marker expression (chromogranin A, synaptophysin, NSE, INSM1); high Ki-67 proliferation index; frequent p53 abnormality and Rb loss in LCNEC PD-L1 expression predominantly in tumor-infiltrating immune cells (TIICs) CD8^+^ TIL density NETest as circulating molecular monitoring tool	Platinum-based chemotherapy (etoposide–platinum or irinotecan–platinum) Definitive chemoradiotherapy in locally advanced disease Immune checkpoint inhibition (PD-1/PD-L1) potentially beneficial in PD-L1^+^/CD8^+^ immune phenotype Palliative systemic chemotherapy for metastatic disease
Adenoid Cystic Carcinoma [71,72,73,74,75]		
Extremely rare (~0.1% of esophageal cancers) Origin from myoepithelial cells and intercalated ducts of submucosal glands	Histology: epithelial cells and myoepithelial cells variably arranged in cribriform, tubular/glandular, and solid architectures. IHC: Epithelial cells variably express cytokeratins, CEA, and CD117; myoepithelial cells variably expression SMA, S100, CK5/6, p40, p63 Molecular: rearrangement involving *MYB*, *MYBL1*, and *NFIB*	KIT/C-KIT pathway EGFR pathway Myoepithelial differentiation pathways
Undifferentiated Carcinoma [76,77,78,79,80,81,82,83,84,85,86,87]		
Rare tumor with male predominance and presentation between 39 and 84 years Arises in lower esophagus or gastroesophageal junction; associated with Barrett’s esophagus Presents with severe weight loss Highly aggressive with frequent metastases to liver, lung, and bone at diagnosis Absence of squamous, glandular, or neuroendocrine differentiation	Morphology: Absence of definitive line of differentiation (i.e., lack of squamous, glandular, or neuroendocrine differentiation); pleomorphic discohesive cells; high mitotic activity, necrosis, rhabdoid features IHC: Suggestive panel could include keratin AE1/AE3, synaptophysin, chromogranin A, BRG1 (SMARCA4), BRM (SMARCA2), INI1 (SMARCB1), and SALL4 Molecular: variable *TP53*, *ARID1A*, *APC*, *CTNNB1* alterations; variable SWI/SNF deficiency	Limited response to conventional chemotherapy; immunotherapy (pembrolizumab) in ARID1A-mutant cases Cadonilimab + anlotinib in SMARCA4-deficient tumors Potential strategies include EZH2 inhibition, etoposide, and combined epigenetic therapy with PD-1 blockade
Gastrointestinal Stromal Tumor [88,89,90,91,92,93,94,95,96,97,98]		
Most common mesenchymal GI neoplasm; ~2% of all GI tumors; esophageal occurrence is rare Clinical risk stratified by anatomic location, tumor size, and mitotic rates	Histology: ~70% spindle-cell, ~20% epithelioid, remainder mixed IHC: DOG1, KIT (CD117) Molecular: Most with either *KIT* or *PDGFRA* mutations, remaining few with alterations in SDH subunit genes, *NF1*, *BRAF,* or *KRAS* LOH at 1p, 14q, 22q (NF2 locus)	KIT pathway PDGFRA pathway BRAF V600E signaling NF2-associated pathways
Gastroblastoma [99,100]		
Rare (~27 reported cases worldwide) Submucosal-like lesion can mimic GIST Pain, bleeding, anemia	Biphasic gastric tumor (epithelial and mesenchymal components) IHC: AE1/AE3, CK7 (epithelial), vimentin, CD10, CD56, nuclear GLI1; negative for CD117, DOG1 GLI1 pathway rearrangements (*MALAT1*::*GLI1* most common)	Hedgehog/GLI1 pathway PD-L1 HDAC2

## Data Availability

No new data were created or analyzed in this study. Data sharing is not applicable to this article.
